# Clinical Scoring Systems to Diagnose Complicated Acute Appendicitis in a Rural Hospital: Are They Good Enough?

**DOI:** 10.7759/cureus.64927

**Published:** 2024-07-19

**Authors:** Padmapriya Balakrishnan, Pratheesh Munisamy, Saravanakumari Vijayakumar, Pammy Sinha

**Affiliations:** 1 Pathology, Sri Lakshmi Narayana Institute of Medical Sciences and Hospital, Pondicherry, IND; 2 General Surgery, Sri Lakshmi Narayana Institute of Medical Sciences and Hospital, Pondicherry, IND

**Keywords:** rural hospital, complicated, alvarado score, clinical prediction scores, acute appendicitis

## Abstract

Introduction

Acute appendicitis (AA) is the most common surgical emergency in developed countries, whose incidence peaks in the second and third decades. The risk of mortality in uncomplicated AA is very low. There are many scoring systems to predict AA. Prediction scores are used less frequently to predict complicated AA. Rural hospitals are often constrained by a lack of round-the-clock imaging or special laboratory services, which may enable accurate diagnosis.

Materials and methods

This study aimed to determine whether prediction scores without imaging or C-reactive protein (CRP) levels could predict complicated AA in a rural setting. All cases of AA for the previous 13 months were recruited for the study. Demographic data, clinical signs and symptoms, complete blood counts, intraoperative findings, and the corresponding histopathological results were collated. The scoring systems (Alvarado, RIPASA, Tzanakis, and Ohmann) were calculated from the clinical and laboratory data. Demographic variables, clinical features, and histopathological findings are described as frequencies/proportions. Chi-squared and Student’s t-tests were used to analyze differences between patients with complicated and uncomplicated AA. A receiver operating curve (ROC) analysis was performed to calculate the area under the curve (AUC) and determine whether appendicitis scores could predict complicated AA.

Results

There were 76 patients with a mean age of 29.1±13.0 years. Serositis was observed in 65% of the patients; mucosal ulceration was the most common microscopic finding, with a pathological diagnosis of AA in 58 (76.3%) patients. Rovsing’s sign and the presence of phlegmon and granuloma were significantly different between those with and without complicated AA. The clinical prediction scores were not significantly different between the two groups. The Tzanakis and Ohmann scores were significant (cutoff: 6.5 and 7.25, p=0.001 and 0.01, respectively) in diagnosing AA (sensitivity/specificity of 98.3/66.7 and 98.3/94.4, respectively). With a cutoff of 5.75, the RIPASA score, with an AUC of 0.663 (p=0.09), showed the highest sensitivity (90.7) and specificity (76.6) for diagnosing complicated AA.

Conclusion

Diagnosing AA based solely on clinical presentation remains a challenge. This study showed that clinical scores such as those of Alvarado, RIPASA, Tzanakis, and Ohmann could not accurately predict complicated AA. Scoring systems without imaging and intraoperative diagnoses are not infallible; therefore, histopathological examination of the resected appendix is mandatory.

## Introduction

Acute appendicitis (AA) is the most common surgical emergency in the developed world [1]. The incidence of acute AA is approximately 5.7-50/100000 inhabitants per year in developed countries [2]. The crude incidence rate in developing countries such as Turkey is 10/1000 [3]. The peak incidence of AA is usually in the second-third decades [4]. It is a disorder afflicting children and young adults in the second and third decades, with a male preponderance [5]. An Iranian study showed a higher incidence in autumn and winter [3]. The perforation rate in AA varies from 16% to 40% [2]. Perforation is common in children and older adults [6]. The risk of mortality in uncomplicated AA is approximately 0.1% and 5% in patients with perforation [2].

Although many scoring systems are available for diagnosing AA, such as Alvarado, Tzanakis, Ohmann, and RIPASA (Raja isteri pengiran anak saleha appendicitis) scores, none are widely acceptable [7]. Among the clinical decision rules, the Alvarado score is the most commonly used [4]. The Alvarado and Appendicitis Inflammatory Response (AIR) score can be used to stratify patients into three groups: low (Alvarado 5-6), intermediate (Alvarado 7-8), and high-risk (Alvarado 9-10) [4]. The AIR score is probably the most pragmatic system for predicting AA [2]. The Adult Appendicitis Score (AAS) was developed by Sammalkorpi et al. in Finland to study the effect of negative appendectomy rates before and after introducing the score in two university hospitals [8]. Prediction scores are used less frequently to predict complicated AA. Scoring systems, such as Appendicitis Severity Index (SAS), APpendicitis Severity Index (APSI), and Atema, have used clinical, biochemical, and CT findings to differentiate complicated and uncomplicated AA [9,10]. Rural hospitals are often constrained by a lack of round-the-clock imaging or special laboratory services, which may enable accurate diagnosis. Hence, this study aimed to determine whether prediction scores without imaging or C-reactive protein (CRP) levels could predict complicated AA in a rural setting.

## Materials and methods

We aimed to determine whether clinical prediction scoring systems could predict intraoperative complicated acute appendicitis among patients in a rural hospital setting. This retrospective study was performed in the Sri Lakshminarayana Institute of Medical Sciences under the auspices of the Departments of Pathology and General Surgery. IEC approval (IEC/C-P/19/2024 dated 08 February 2024) was obtained before commencing the study. A list of all cases of acute appendicitis that were operated on in the Department of General Surgery between 01 January 2023 and 27 February 2024 was obtained from the departmental operation theater registers. Using the case sheet numbers, copies of the files were obtained from the Medical Records Department. Biopsy numbers with a diagnosis of acute appendicitis were obtained from the Department of Pathology and with the matching data from the case files, slides were reviewed by the Pathology coauthors. Demographic data, clinical signs and symptoms, complete blood counts, ultrasonogram (USG), intraoperative findings, and clinical prediction scores were entered into an Excel spreadsheet (Microsoft Corporation, Redmond, WA) by the first two authors. Scoring systems (Alvarado, RIPASA, Tzanakis, and Ohmann) were calculated from the available clinical and laboratory data. Corresponding histopathological findings were collated into the spreadsheet, and the curated data were analyzed using Statistical Product and Service Solutions (SPSS, version 23; IBM SPSS Statistics for Windows, Armonk, NY). Demographic variables, clinical features, radiological findings, intraoperative findings, and histopathological findings were described as frequencies/proportions. Continuous data such as age and pulse rate were described as means±SD. Medians with interquartile ranges were calculated for the prediction scores. The chi-squared and Student's t-test were used for discrete and continuous data, respectively, to analyze the differences between those with complicated and uncomplicated acute appendicitis. A receiver operating curve analysis (ROC) was performed to calculate the area under the curve (AUC) to determine whether the appendicitis scores could predict complicated acute appendicitis. In the ROC calculation, the diagnosis of acute appendicitis and complicated AA were used as state variables, with all four prediction scores (Alvarado, RIPASA, Ohmann, and Tzanakis) used as test variables.

## Results

There were 76 patients with a nearly equal proportion of sexes, with a mean age of 29.1±13.0 years (Table 1).

**Table 1:** Baseline demographics, clinical features, and lab data RIF - right iliac fossa; WBC - white blood cell count; RIPASA - Raja isteri pengiran anak saleha appendicitis

	Characteristic of 76 patients	Means±SD; N (%)
Demographics	Age(years)	29.1±13.0
	Females(n)	42 (55.3)
Symptoms	Duration of symptoms (<48h) (n)	43 (56.6)
	Vomiting (n)	44 (57.9)
	Anorexia (n)	35 (46.1)
	Continuous pain RIF (n)	75 (98.7)
	Migratory pain (n)	19 (25)
	Fever (n)	29 (38.2)
	Constipation (n)	7 (9.2)
	Urinary symptoms (n)	9 (11.8)
Signs	RIF tenderness (n)	75 (98.7)
	Rebound tenderness (n)	50 (65.8)
	Guarding (n)	19 (25)
	Rigidity (n)	7 (9.2)
	Rovsing sign (n)	14 (18.4)
Lab tests	WBC> 10x10^9^/L (n)	45 (59.2)
	Neutrophilia, shift to the left (n)	39 (51.3)
Scoring system - Median/IQR		
	Alvarado score	6 (4-7)
	Ohmann score	11.25 (9.5-12.5)
	Tzanakis score	13 (10-13)
	RIPASA score	7.5 (6-9.5)

The most common clinical signs and symptoms were persistent pain in the right iliac fossa and tenderness. The appendix was visualized in 3/4ths of the patients on USG (Table 2).

**Table 2:** Ultrasonographic, intraoperative, and histopathological findings

	Characteristics of the 76 patients	N (%)
USG findings	Sonographic findings suggestive(n)	63 (82.9)
	Visualised appendix (n)	58 (76.3)
	Width >6mm (n)	51 (67.1)
	Fecolith (n)	9 (11.8)
	Wall thickening >3mm (n)	26 (34.2)
	Cecal apex thickening (n)	11 (14.5)
	Periappendiceal inflammation (n)	35 (46.1)
	Fat stranding (n)	59 (77.6)
	Extraluminal fluid (n)	23 (30.3)
	Abscess (n)	5 (6.60
	Gas (n)	1 (1.3)
	Necrosis (n)	1 (1.3)
Surgery	Laparoscopy (n)	57 (75)
	Retrocecal position of appendix (n)	49 (64.5)
	Purulent fluid (n)	18 (23.7)
	Phelgmon/ adhesions (n)	39 (51.3)
	Intrabdominal abscess (n)	6 (7.9)
	Necrosis (n)	4 (5.3)
	Perforation (n)	5 (6.6)
Histopathology	Swollen appendix (n)	56 (73.7)
	Serositis (n)	50 (65.8)
	Exudate (n)	12 (15.8)
	Gangrene (n)	6 (7.9)
	Perforation (n)	1 (1.3)
	Fecolith (n)	7 (9.2)
	Exudate (n)	33 (43.4)
	Mucosal ulceration (n)	54 (71.1)
	Hyperplastic follicle (n)	13 (17.1)
	Neutrophilic infiltrate (n)	37 (48.7)
	Eosinophilic infiltrate (n)	24 (31.6)
	Granuloma (n)	1 (1.3)
	Peri appendicitis (n)	24 (31.6)
	Acute appendicitis (n)	58 (76.3)
Alvarado score	Low risk 5-6	27 (35.6)
	Intermediate-risk 7-8	20 (26.3)
	High-risk 9-10	7 (9.2)

**Figure 1:**
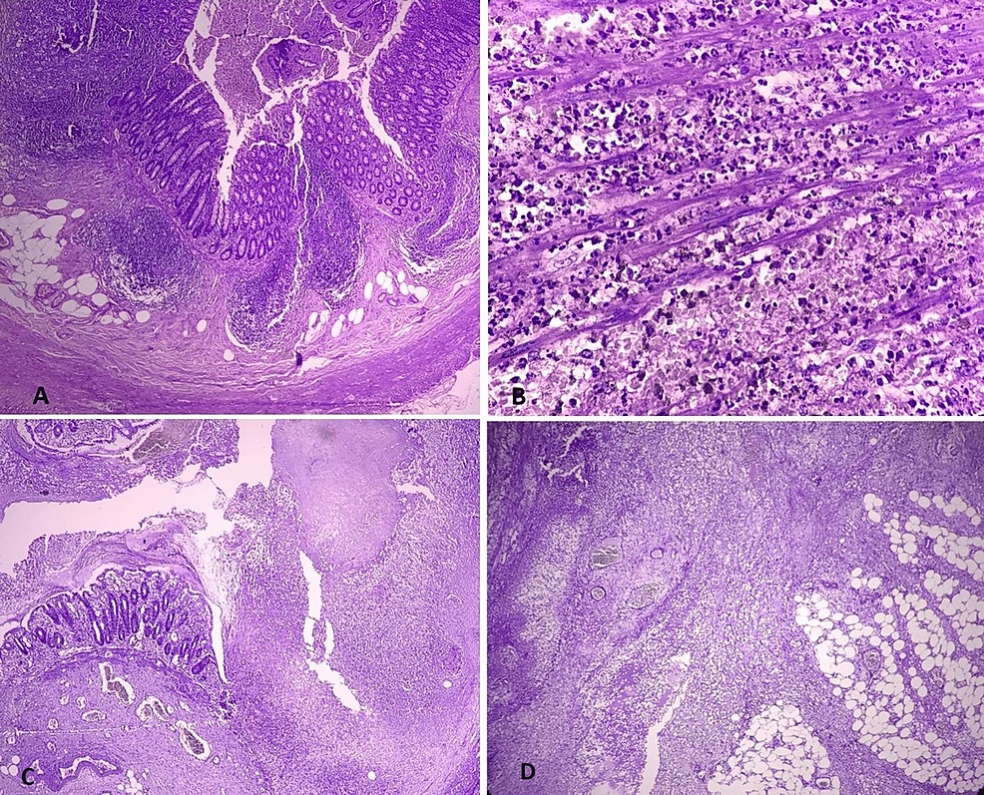
Histopathological examination of acute appendicits 1A: Hematoxylin-eosin (HE) stains 4X - Early appendicitis with surface mucosal ulceration 1B: HE stains, 40X - Transmural neutrophilic infiltrate 1C: HE stains, 4X - Acute suppurative appendicitis with mural necrosis 1D: HE stains, 4X - Periappendicitis (neutrophilic infiltrate extending to muscularis propria, serosa, and periappendicular adipose tissue with congested blood vessels)

Serositis was observed in 65% of the patients; mucosal ulceration was the most common microscopic finding, with a pathological diagnosis of acute appendicitis in 58 (76.3%) patients (Figures 1A-1C). The remaining 18 patients had resolving appendicitis (n=4), lymphoid hyperplasia (n=9), mucoceles (n=1), fibrous obliteration (n=2), granulomatous (n=1), and normal histology (n=1).

There were some discrepancies in the intraoperative findings compared with the pathological diagnosis: perforation (5 vs. 1) and gangrene (4 vs. 6). Rovsing’s sign and the presence of phlegmon and granuloma were significantly different between those with and without complicated acute appendicitis. The clinical prediction scores were not significantly different between the two groups (Table 3).

**Table 3:** Comparison between complicated and uncomplicated appendicitis RIF - right iliac fossa; WBC - white blood cells; USG - ultrasonogram; ICU - intensive care unit; RIPASA - Raja isteri pengiran anak saleha appendicitis

Characteristic	Uncomplicated (n=47)	Complicated (n=11)	P values
Age (y)	30.0±13.9	27.9±13.6	0.65
Gender Males (n)	20 (42.6)	8 (72.7)	0.07
Symptoms> 48 h (n)	22 (46.8)	4 (36.4)	0.53
Vomiting (n)	23 (48.9)	8 (72.7)	0.15
Anorexia (n)	17 (36.2)	7 (63.6)	0.09
Continuous RIF pain (n)	47 (100)	11 (100)	-
Migratory pain (n)	11 (23.4)	1 (9.1)	0.28
Fever (n)	18 (38.3)	7 (63.6)	0.12
Urinary complaints (n)	7 (14.9)	1 (9.1)	0.61
Pulse rate (beats/min)	84.7±10.6	91.3±16.1	0.08
Rebound tenderness (n)	36 (76.6)	8 (72.7)	0.78
Guarding (n)	10 (21.3)	5 (45.5)	0.09
Rigidity (n)	5 (10.6)	2 (18.2)	0.48
Rovsing sign (n)	8 (17)	5 (45.5)	0.04
WBC >10x10^9^/L (n)	26 (55.3)	6 (54.5)	0.96
Neutrophilia (n)	27 (57.4)	6 (54.5)	0.86
USG findings (n)	44 (93.6)	9 (81.8)	0.29
Laparoscopy (n)	41 (87.2)	4 (36.4)	0.001
Phlegmon (intraoperative)(n)	22 (46.8)	10 (90.9)	0.008
Antibiotic change (n)	4 (8.5)	4 (36.4)	0.016
Need for ICU care (n)	3 (6.4)	1 (9.1)	0.75
Length of stay (d)	5.6±1.4	7.0±2.3	0.17
Alvarado	5.9±2.0	6.1±2.1	0.70
RIPASA	7.8±2.4	9.4±2.6	0.06
Ohmann	11.3±2.0	11.0±1.54	0.75
Tzanakis	12.2±2.0	11.6±2.8	0.37
Histopathology			
Swollen appendix (n)	39 (83)	7 (63.6)	0.15
Serositis (n)	36 (76.6)	9 (81.8)	0.70
Exudate (n)	7 (14.9)	5 (45.5)	0.02
Fecolith (n)	7 (14.9)	0	0.17
Exudate (n)	22 (46.8)	8 (72.7)	0.12
Mucosal ulceration (n)	42 (89.4)	9 (81.8)	0.48
Hyperplastic follicle (n)	5 (10.6)	1 (9.1)	0.87
Neutrophilic infiltrate (n)	29 (61.7)	8 (72.7)	0.49
Eosinophilic infiltrate (n)	18 (38.3)	3 (27.3)	0.43
Granuloma (n)	0	1 (9.1)	0.03
Peri appendicitis (n)	18 (38.3)	5 (45.5)	0.66
Alvarado score-based risk stratification			
Low-risk (n)	11 (23.4)	3 (27.3)	0.78
Intermediate-risk (n)	17 (36.2)	4 (36.4)	0.99
High-risk (n)	19 (40.4)	4 (36.4)	0.80

**Figure 2:**
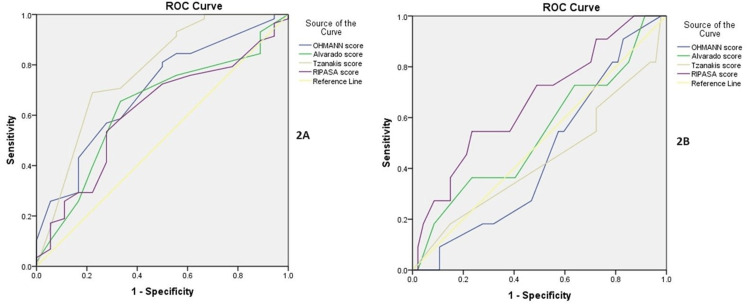
Receiver operating characteristic curves for the diagnosis of acute appendicitis (2A) and complicated acute appendicitis (2B)

Clinical risk stratification based on the Alvarado score showed a negative appendectomy rate of 15% in high-risk individuals and 36.4% in low-risk individuals; the overall negative appendectomy rate was 23% (Table 1). The Tzanakis and Ohmann scores were significant (cutoff: 6.5 and 7.25, p=0.001 and 0.01, respectively) in diagnosing acute appendicitis (sensitivity/specificity of 98.3/66.7 and 98.3/94.4, respectively). With a cutoff of 5.75, the RIPASA score, with an AUC of 0.663 (p=0.09), showed the highest sensitivity (90.7) and specificity (76.6) for diagnosing complicated acute appendicitis (Figures 2A-2B). Regression analysis showed that the predictors for complicated AA included pulse rate (p=0.01), Rovsing’s sign (p=0.04), cecal apex thickening (p=0.005), extraluminal fluid (p=0.04), abscess (p<0.001), and gas (p=0.03) on USG.

## Discussion

The incidence of appendicitis is increasing in India, probably because of urbanization and dietary changes [11]. Scoring systems (clinical and imaging) have enabled conservative management of low-risk patients in developed countries. In surgical practice, the appendix remains the most commonly resected organ [6]. Surgery remains the primary mode of therapy for AA in developing countries. Rural poverty, geographical location, and transportation affordability are related to delays in arrival and surgery [12]. In a study by Nabipour, 76.6% of patients were less than 30 years old, while this subset constituted 51.5% of patients in the current study [3]. In South Africa, among 200 patients, the average age in males and females was 21.5 and 22.2 years [1]. The corresponding ages in this study were 26.9 and 30.9 years, respectively.

The pathological classification of AA has been classified differently in different studies: early acute appendicitis, acute focal appendicitis, acute suppurative appendicitis, gangrenous appendicitis, and perforated appendicitis by Nabipour [3]; acute suppurative appendicitis (including gangrene and perforation), early appendicitis, and periappendicitis by Thanaletchimy [13]; borderline AA (intraluminal, mucosal, or submucosal inflammation); phlegmonous AA; and complicated AA by Hoffman et al. [14]. AA could be classified as acute intraluminal inflammation, acute mucosal inflammation, acute mucosal and submucosal inflammation, suppurative AA, gangrenous AA, periappendicitis, and increased mural eosinophils, a classification that has been used in this study [15].

Complicated AA is defined as “AA with periappendicular contained phlegmon or purulent/free fluid (type 2a), AA with an intraabdominal abscess (type 3a), gangrenous AA with (type 3b) or without (type 2b) perforation" [14]. Uncomplicated AA is classified as borderline AA (type 1a) and phlegmonous AA (type 1b) [14,16]. The stratified clinical approach to AA to effectively manage such patients classifies acute appendicitis into normal appendix (normal, acute intraluminal inflammation, acute mucosal inflammation), simple, non-perforated appendix (suppurative/phlegmonous), and complex appendicitis (gangrenous, perforated, and abscess) [4]. Ten percent had complicated AA compared with 18.9% (n=11/58) in the current study [8].

Many prediction scores have been used for the diagnosis of AA. Very few studies have been conducted on the diagnosis of complicated AA. The Alvarado score does not have adequate specificity, and in elderly patients, it cannot differentiate complicated AA [2]. Based on the Alvarado score, the patients in the current study were stratified into low-risk (n=22), intermediate-risk (n=27), and high-risk (n=27) groups [4]. The RIPASA score was found to be more useful in Asian populations than the Alvarado score, but we did not find it to be accurate enough (71% vs. 81.5% for Tzanakis) [2]. The median Alvarado, Ohmann, RIPASA, and Tzanakis scores in a Turkish study were 7, 13, 10, and 13, respectively [7]. The corresponding values in the present study were 6, 11.25, 7.5, and 13. The Tzanakis score was the best predictor of AA in both studies, while RIPASA was not useful in the current study (p=0.14, AUC =0.614. Using AAS to risk-stratify patients results in a low negative appendectomy rate (NAR) [2]. The NARs vary between nine and 27.3% in Asia and Africa; the rate in this study was 23%. NARs are higher in women, ranging from 20% and 45%; and a review in 2000 reported that these NARs have remained unchanged over the past 70 years [15]. Among the 249 patients from Australia (M=113 and F=136), the NAR was 9.7% in males and 39% in females [17]. The Australian study reported an overall NAR of 25% [17]. Another study from Bangalore reported an NAR of 9% among 230 specimens [11]. The NAR was 8.7% in a Finnish study [8]. The high-risk group will require CT imaging before surgery according to the AAS [8]. CT was not routinely performed for our patients. We could not use either AIR or AAS because of the unavailability of CRP in all the patients. Among the 15 prediction models, AAS performed best for women, while AIR performed best in men [2].

Pregnant women with nausea, vomiting, and elevated WBC count may have lower accuracy of these scoring systems [2]. Biomarkers that have been used in AA include CRP, calprotectin, procalcitonin, and the APPY test panel [2]. Complicated AA is often associated with higher WBC and CRP levels [2]. Older age, female sex, duration >48 hours, and elevated CRP levels are risk factors for complicated AA [18]. More than 20% of appendectomies are associated with complications [18]. In a study from Jeddah, complicated AA had shorter pain duration, higher neutrophil percentage, elevated WBC, higher CRP, larger appendix diameter, and free fluid on imaging compared to simple uncomplicated AA [18]. None of these features was significant in the current study. A total of 18.9% had complicated AA compared to 13.4% in the Jeddah study [18]. A Chinese study by Kang et al. used flow cytometry to determine CD3, CD4, CD8, CD16, CD19, and CD56 [19]. Significant differences between simple AA and purulent AA were observed in CD4 cells, T cells, B cells, and the CD4/CD8 ratio [19].

The risk of perforation generally increases after 48 hours and may be as high as 40-60% in developing and underdeveloped countries [12]. Perforation in a study of 240 specimens from Bahrain was observed in 1.17% (n=4) [20]. Younger children have rates of perforation reaching 40-57% [21]. Eight percent of our study population were adults. Twelve of the 15 children had pathological AA. The only perforation observed in the study was a four-year-old male child. Gangrene and perforation were reported in 2.2% and 1.3% of the cases, respectively, in a study from Bangalore [11]. Over a period of five years (n=2753), 221 (8%) were gangrenous, and 21 (0.8%) were perforated appendices [3]. The corresponding percentages in the current study were 7.9 and 1.3. Perforation is generally higher among patients in rural areas [4]. There was only one case of perforation in the study population.

Very few studies have been conducted on rural populations. A study in rural Tibet compared complicated and uncomplicated AA in 93 patients [22]. Patients with complicated AA had significant fever, elevated WBC and CRP levels, and a higher modified Alvarado score [22]. The current study did not significantly predict complicated AA using the four prediction models. They studied AIR, AAS, RIPASA, and modified Alvarado scores. The AIR score showed the best performance with 77.8% sensitivity and 75% specificity [22]. Age, sex, and duration of illness were independent risk factors for complicated AA [22].

In another rural study from Turkey, prospective data were collected over two years in 151 patients. USG could be done only in 78 [12]. Referrals from remote towns and referrals during the winter season were predictors of complicated AA. Females constituted 27.8%, compared to 53% in the current study [12]. In our study population, 64.5% had a low-risk Alvarado score of 5-6 compared to 7.9% in the Turkish study [12]. We did not study the seasonal influence on the incidence of AA.

The diagnostic workup may vary between countries and institutions based on prediction scores and imaging [9]. This hospital performed clinical examinations and USG imaging for all patients with suspected AA. USG was suggestive in 63 patients (83.9%), yielding a sensitivity and specificity of 91.3% and 44.5%, respectively, compared with 69% and 81% in the literature [9]. There are no clear guidelines for differentiating between complicated and uncomplicated AA. We could not perform SAS or APSI scoring because of the lack of CRP. There is a definite role for nonoperative management in patients with a low risk of AA and, hence, the need to differentiate between complicated and uncomplicated AA [9]. The availability of CT and its newer techniques makes CT a viable option for all scoring systems to reduce NAR and differentiate uncomplicated and complicated AA [9].

Imaging is generally not recommended in low-risk patients (Alvarado) because of the high yield of false-positive results [23]. USG was performed on all patients according to institutional policy. A cutoff <5 can exclude most patients with AA, but 14/22 (63.6%) patients with an Alvarado score <5 had AA on pathological examination in the current study [23]. Approximately 90% of patients with high-risk scores (Alvarado) had AA [23]; correspondingly, in this study, 85.7% (6/7) of high-risk patients and 85.2% (17/20) of patients in the intermediate-risk group had confirmed AA. A 6 mm cutoff for the outer dimension of the appendix on USG is 100% sensitive and 68% specific for AA [21]. Using a similar cutoff, the current study revealed only 77.5 sensitivity and 66.6% specificity.

Some studies reported a large proportion of worm infestations (n=74/1000) and carcinoid tumors (2/1000) in their case series [13]. The current study did not report worms or tumors. Normal histology was observed in only one patient, compared to 5.7% in a Malaysian study [13]. In the present study, periappendicitis and eosinophilic infiltrates were observed in 31.6% of the patients (Figure 1D). Periappendicitis was observed in 1.4% of Malaysian study [13]. Abdominopelvic inflammation due to ovarian neoplasms, gastrointestinal carcinoma, inflammatory bowel disease, pelvic inflammatory disease, and urologic disorders can cause inflammation of the appendiceal surface [15]. In the current study, periappendicitis was observed in 83.3% of gangrenous AA (p=0.004), 100% of perforations, 42.9% of fecoliths, 57.4% of those without mucosal ulcerations (p=0.001), 15.4% of hyperplastic follicles, and 59.5% of neutrophilic infiltrates (p<0.001).

Limitations

CRP could not be performed in all patients because this test was performed only during routine working hours. This was a single-center retrospective cohort study. Since it is a private medical college hospital, unaffordable patients may have traveled 10 km (20-25 min) to the nearest public health institution in the city for surgical services.

## Conclusions

Diagnosing acute appendicitis based on clinical presentation remains a challenge, with a negative exploration rate of up to 25%. Avoidable procedures put patients at risk, increase complications, and delay necessary treatment. This study showed that clinical scores such as those of Alvarado, RIPASA, Tzanakis, and Ohmann could not accurately predict complicated acute appendicitis. Scoring systems without imaging and intraoperative diagnoses are not infallible; therefore, histopathological examination of the resected appendix is mandatory. Scoring systems that use CT are required to reduce NAR and avoid delays in surgery.
